# Multi-Factors Cooperatively Actuated Photonic Hydrogel Aptasensors for Facile, Label-Free and Colorimetric Detection of Lysozyme

**DOI:** 10.3390/bios12080662

**Published:** 2022-08-20

**Authors:** Peiyan Shen, Yuqing Shi, Ran Li, Bo Han, Haojie Ma, Xueyan Hou, Yuqi Zhang, Lei Jiang

**Affiliations:** 1 Key Laboratory of New Energy and New Functional Materials, Shaanxi Key Laboratory of Chemical Reaction Engineering, College of Chemistry and Chemical Engineering, Yan’an University, Yan’an 716000, China; 2 Technical Institute of Physics and Chemistry, Chinese Academy of Sciences, Beijing 100190, China

**Keywords:** photonic hydrogel aptasensors, multi-factors actuation, aptamer conformational change, volume phase transition, colorimetric sensing

## Abstract

Responsive two-dimensional photonic crystal (2DPC) hydrogels have been widely used as smart sensing materials for constructing various optical sensors to accurately detect different target analytes. Herein, we report photonic hydrogel aptasensors based on aptamer-functionalized 2DPC poly(acrylamide-acrylic acid-N-tert-butyl acrylamide) hydrogels for facile, label-free and colorimetric detection of lysozyme in human serum. The constructed photonic hydrogel aptasensors undergo shrinkage upon exposure to lysozyme solution through multi-factors cooperative actuation. Here, the specific binding between the aptamer and lysozyme, and the simultaneous interactions between carboxyl anions and N-tert-butyl groups with lysozyme, increase the cross-linking density of the hydrogel, leading to its shrinkage. The aptasensors’ shrinkage decreases the particle spacing of the 2DPC embedded in the hydrogel network. It can be simply monitored by measuring the Debye diffraction ring of the photonic hydrogel aptasensors using a laser pointer and a ruler without needing sophisticated apparatus. The significant shrinkage of the aptasensors can be observed by the naked eye via the hydrogel size and color change. The aptasensors show good sensitivity with a limit of detection of 1.8 nM, high selectivity and anti-interference for the detection of lysozyme. The photonic hydrogel aptasensors have been successfully used to accurately determine the concentration of lysozyme in human serum. Therefore, novel photonic hydrogel aptasensors can be constructed by designing functional monomers and aptamers that can specifically bind target analytes.

## 1. Introduction

Responsive two-dimensional photonic crystal (2DPC) hydrogels, as smart sensing materials, have attracted increasing attention due to the Debye diffraction effect of 2DPC, volume phase transition of hydrogels and facile functionalization by recognition agents [1,2]. The interaction between recognition agents and target analytes induces the hydrogel volume to change, altering the particle spacing of the 2DPC embedded in the hydrogel network. The concentration of the target analytes can be determined by monitoring the particle spacing change of the 2DPC hydrogel sensor. The particle spacing can be acquired from measuring the diameter of Debye ring diffracted by 2DPC, in which special instruments are not needed and a simple laser pointer is enough [3]. In recent years, many sensors based on 2DPC hydrogels functionalized by various recognition agents have been developed for label-free detection of small biological molecules [4,5,6,7], proteins [8,9], drugs [10] and microorganisms [11,12]. For example, Asher’s group and Meng’s group fabricated organic molecules functionalized 2DPC hydrogels for the detection of phenylalanine [4], glucose [5,6] and concanavalin A [8]. Cai et al. and Wu et al. modified carbohydrates, proteins and enzymes into 2DPC hydrogels to detect lectin proteins including ricin and jacalin [9], candida albicans [11], H_2_O_2_ [13], urea and phenyl phosphorodiamidate [10]. Among the recognition agents, aptamers, which are single-stranded DNA or RNA oligonucleotides, are widely used for constructing sensors due to the simple synthesis, easy modification, high chemical stability and the high specificity and affinity to target analytes [14,15]. The aptamer-based sensors have been developed for detecting various analytes [16,17,18,19,20,21,22,23,24,25,26], such as metal ions [19,20,21], antibiotics [22,23], proteases [24,25] and biomarkers [26]. Several aptamer-based photonic hydrogel sensors have also been reported. For example, Gu’s group [27] first constructed a Hg^2+^-sensitive three-dimensional inverse opal photonic crystal hydrogel functionalized by an aptamer for colorimetric detection of Hg^2+^. Recently, several aptasensors based on 2DPC hydrogels have been used to achieve the detection of cysteine [28,29], adenosine [30] and SARS-CoV-2 [31]. Therefore, it is a promising strategy to construct various sensors via combining aptamers with 2DPC hydrogels, based on the specific binding between aptamers and analytes, Debye diffraction of 2DPC and the volume phase transition of hydrogel, achieving simple, low-cost and selective detection of target analytes.

Lysozyme is a native protein with ellipsoidal structure due to the interaction of hydrophobic groups [32]. The lysozyme level in body fluid is closely related to leukemia [33], renal disease [34], meningitis [35] and other diseases [36]. For example, the increase of lysozyme concentration in urine may be caused by renal tubular cell damage [37]. The concentration increase in serum may be caused by monocytic leukemia [33]. Therefore, developing a simple lysozyme biosensor is of great significance for the clinical diagnosis for some diseases.

At present, many sensors have been developed for detecting lysozyme using different techniques [38,39,40,41,42,43,44], such as surface-enhanced Raman spectroscopy [38], colorimetric [39], fluorometric [40,41], electrochemical method [42] and enzyme-linked immunosorbent assay (ELISA) [43]. For example, Dhakal and Sapkota [41] fabricated an aptasensor based on fluorescence resonance energy transfer for the detection of lysozyme with a limit of detection (LoD) of 30 nM. Zhao and coworkers [44] reported a magnetoelastic immunosensor by immobilizing an antilysozyme antibody into a magnetoelastic chip to detect lysozyme. However, the reported lysozyme-sensitive sensors need specific instruments [38], fluorophore-labeled aptamers [41], unstable and high-cost antigens [44] or show unstable sensing signals [42]. Therefore, it is still necessary to explore new methods and materials to achieve the simple, accurate and reliable detection of lysozyme.

Herein, we constructed a diamino-terminated aptamer modified 2DPC poly(acrylamide-acrylic acid-N-tert-butyl acrylamide) hydrogel, P(AAm−AAc−TBAm), for facile, label-free and colorimetric detection of lysozyme. The multi-factors, including the specific binding between the aptamer and lysozyme, the electrostatic interaction of carboxyl anions and lysozyme, the interaction of the N-tert-butyl group and the hydrophobic residues on the surface of lysozyme, cooperatively actuated photonic hydrogel aptasensors to shrink. The lysozyme-induced hydrogel shrinkage was expressed by the particle spacing decrease of the 2DPC embedded in the hydrogel network, which can be acquired by measuring the Debye diffraction ring diameters of the photonic hydrogel aptasensors using a laser pointer and a ruler. Compared with antibodies, the constructed 2DPC hydrogel-based aptasensors used stable and low-cost aptamers as specifically binding recognition agents to improve the detection selectivity [40,41]. Furthermore, the Debye diffraction effect of 2DPC has been utilized to achieve a facile detection without needing sophisticated apparatuses.

## 2. Experimental Methods

### 2.1. Materials and Characterization

Lysozyme (≥20,000 untis/mg), hemoglobin, trypsin and the amino-terminated aptamer, 5′−NH_2_− (CH_2_)_6_−ATCTACGAATTCATCAGGGCTAAAGAGTGCAGAGTTACTTAG− (CH_2_)_6_−NH_2_−3′, were acquired from Sangon Biotech. Co., Ltd. (Shanghai, China). Human albumin, bovine serum albumin and adenosine were purchased from Sigma-Aldrich Co., Ltd. (Saint Louis, MO, USA). Adenosine 5-triphosphate disodium salt was purchased from Tansoole Co., Ltd. (Shanghai, China). Acrylamide (AAm), acrylic acid (AAc), N-tert-butyl acrylamide (TBAm), N, N′-methylenebisacrylamide (Bis), 2-hydroxy-4′-(2-hydroxyethoxy)-2-methyl-propiophenone (Irgacure 2959) and 1-ethyl-3-(3-dimethy-laminopropyl) carbodiimide hydrochloride (EDC) were supplied by Aladdin Chemistry Co., Ltd. (Shanghai, China). N-hydroxysuccinimide (NHS) was purchased from J&K Scientific Co., Ltd. (Beijing, China). Human serum was provided by Hefei Bomei Biotech. Co., Ltd. (Hefei, China). AAc was purified by distillation prior to use. An amount of 10 mM of phosphate-buffered saline (PBS) solutions at pH 7.40 were used for all preparation of solutions and measurements. The nanopure water (18.2 mΩ∙cm) was used in all experiments.

The polystyrene (PS) microspheres with the particle diameters at ~960 nm and ~698 nm were synthesized according to the reported method in the literature [45], and then dispersed in water to form latex. PS microspheres with a diameter of ~500 nm were purchased from Shanghai Huge Biotechnology Co., Ltd. (Shanghai, China). 

The microstructures of 2DPC and the hydrogels were observed using a scanning electron microscope (SEM, JSM-7610F, JEOL, Tokyo, Japan) after sputtering gold. Fourier Transform Infrared Spectra (FTIR, Nicolet iS5, Waltham, MA, USA) were used to characterize the compositions of the prepared hydrogels before and after modification of aptamer.

### 2.2. Preparation of the Photonic Hydrogel Aptasensors

**Figure 1:**
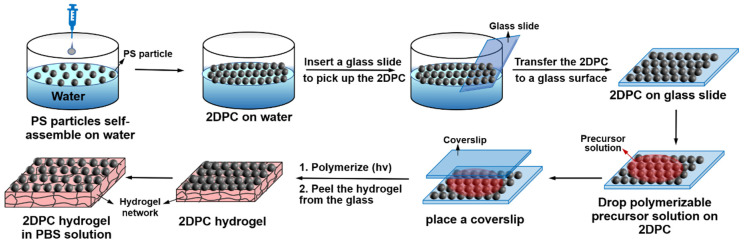
Fabrication of photonic hydrogels. PS particles were injected onto the water surface and orderly self-assembled to form a 2DPC array on water, followed by transfer to a glass slide. Then, polymerizable precursor solution was added to the 2DPC surface on glass substrate, and a coverslip was subsequently placed on the solution. The photopolymerization was performed and the 2DPC was embedded in the hydrogel network. The resultant 2DPC hydrogel was peeled off the glass slide and washed by water and PBS solution, and finally stored in PBS solution.

**Scheme 1:**
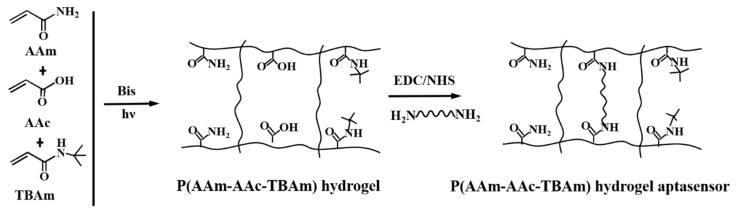
Photopolymerization reaction of monomers and the functionalization of aptamers to fabricate the photonic hydrogel aptasensors.

The lysozyme-sensitive 2DPC hydrogel aptasensors were fabricated by preparing 2DPC hydrogels and subsequently linking the lysozyme-binding aptamers to the hydrogel network. First, the 2DPC hydrogels were fabricated by UV photopolymerization of functional monomers in 2DPC arrays prepared by the needle tip flow method. Figure 1 shows a typical approach for preparing a 2DPC hydrogel. The mixture containing PS microspheres latex and 1−propanol (3:1, *v*/*v*) was carefully injected onto a water surface through a syringe [46]. The PS microspheres diffused and self-assembled into an orderly close-packed 2DPC array on the water surface, followed by transferring to a glass surface (2.4 × 7.6 cm^2^) and drying in air. Then, 110 μL of polymerizable precursor solution (Table 1) was added to the surface of a 2DPC array and a coverslip (2.4 × 3.2 cm^2^) was gently placed on the solution. The photopolymerization reaction (Scheme 1) was performed under UV light of 365 nm for 40 min, which embedded the PS 2DPC into the P(AAm−AAc−TBAm) hydrogel network to form a 2DPC hydrogel. The resultant 2DPC hydrogel was peeled off the glass slide and immersed in nanopure water for 6 h (the water was changed every 1 h), followed by immersion in freshly prepared PBS solution for 6 h (the PBS solution was changed every 1 h). Finally, the 2DPC hydrogel was stored in PBS solution for more than 24 h to reach volume swell equilibrium.

The resultant 2DPC hydrogel was functionalized by the aptamers with a specific bases sequence to acquire a lysozyme-sensitive photonic hydrogel aptasensor. The amino-terminated aptamer was linked to the carboxyl-rich polymer chains of the hydrogel network by forming amide bonds. In detail, the prepared 2DPC hydrogel was cut into pieces with a size of 1 × 1 cm^2^. A piece of 2DPC hydrogel was activated by EDC (15 mg) and NHS (3 mg) [10,27] for 1 h in 1 mL of PBS solution, and then washed with PBS 3 times (2 min was needed for each time). Next, 20 μL of 100 μM aptamer solution was dropped onto the 2DPC hydrogel surface. The modification of aptamer was carried out at room temperature for 1 h, and then at 4 °C overnight. The aptamer-modified 2DPC hydrogel was immersed in PBS solution for 1 h to remove the unreacted aptamers and the washing was repeated 6 times, followed by storing in PBS solution for 24 h before use. Different concentrations of aptamer-functionalized 2DPC hydrogel sensors based on 960 nm PS microspheres were also prepared using the same method.

### 2.3. Measurement of Debye Diffraction Ring

The particle spacing change of a photonic hydrogel aptasensor was acquired by measuring the Debye diffraction ring diameter. The Debye ring diffracted by 2DPC embedded in the hydrogel network can be displayed on a bottom screen by illumination using a laser light along the hydrogel surface, as shown in Figure S1a.

Figure S1b shows the measurement of the Debye ring diameter. The diffraction angle *α* depends on the Debye ring diameter, *D*, and the distance between the 2DPC array and the bottom screen, *h*. The particle spacing of the 2DPC hydrogel can be calculated according to a formula$\;d=4\lambda \sqrt{{\left(D/2\right)}^{2}+h^{2}}/\left(\sqrt{3}D\right)$ [3]. In this study, *λ*, the laser light wavelength, was 532 nm, and *h* was set at 116 mm. For each particle spacing measurement, three 2DPC hydrogel aptasensor samples were used, and *d* of each sample was measured at 3 different positions. From the 9 measurements, we can calculate the average of *d* and the standard deviation (*s*) according to a formula $s=\sqrt{\frac{1}{N-1}\sum_{i=1}^{N}{\left(x-\bar{x}\right)}^{2}}$. Therefore, it is just needed to measure the Debye ring diameter using a commercial ruler with millimeter accuracy, followed by calculation of *d* and finally acquirement of the particle spacing change, Δ*d*.

## 3. Results and Discussion

### 3.1. Preparation and Characterization of the Photonic Hydrogel Aptasensors 

The lysozyme-sensitive photonic hydrogel aptasensor was prepared by the radical photopolymerization reaction of monomer solution containing AAm, AAc and TBAm, which was allowed to proceed in a 2DPC array, and the subsequent functionalization of the aptamer (Scheme 1). Therefore, the resultant photonic hydrogel aptasensor was a polymer network with a polyacrylamide backbone, rich carboxyl groups and hydrophobic N-tert-butyl amide groups. After activation by EDC/NHS, amino-terminated aptamers were covalently linked to the polymer chains of the carboxyl-containing 2DPC hydrogel by formation of amide bonds, acquiring a lysozyme-sensitive photonic hydrogel aptasensor.

**Figure 2:**
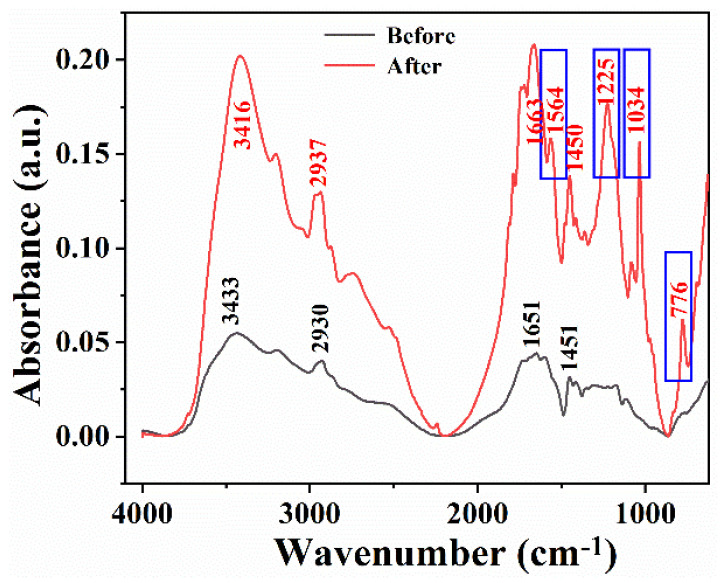
FTIR spectra of the P(AAm−AAc−TBAm) hydrogel before and after modification of lysozyme-binding aptamer. The appearance of new absorption peaks at 1564 cm^−1^, 1225 cm^−1^, 1034 cm^−1^ and 776 cm^−1^ demonstrated the successful modification of the aptamers.

To demonstrate the covalent attachment of lysozyme-binding aptamers to the 2DPC hydrogel, hydrogels without 2DPC were prepared by photopolymerization reaction using the polymerizable precursor solution containing AAm, AAc and TBAm, and then modified by the aptamer. The hydrogel films before and after modification of the aptamers were characterized by FTIR spectra, as shown in Figure 2. For the hydrogel that was modified by the aptamers (red line), the peaks at 3146 cm^−1^, 2937 cm^−1^ and 1663 cm^−1^ represented the stretching vibration of N−H, saturated C−H, and C=O bonds, respectively. The peak at 1450 cm^−1^ can be attributed to the C−H bending vibration of tert-butyl. It also can be found that new absorption peaks appeared at 1564 cm^−1^, 1225 cm^−1^, 1034 cm^−1^ and 776 cm^−1^ compared with the hydrogel before modification of the aptamer. The peak at 1564 cm^−1^ can be attributed to the vibrational absorption of C=C and C=N of DNA in the base planes [47]. The peaks at 1225 cm^−1^ and 1034 cm^−1^ were the vibrational absorption of P=O and C−O bonds and in DNA backbone, respectively [48,49], and 776 cm^−1^ derived from the ring breathing of DNA [50]. The results indicated that the aptamers were successfully modified onto the hydrogel polymer chains. Herein, to avoid interference from infrared absorption peaks generated by the PBS solution and the benzene rings of PS microspheres, ultrapure water was used to prepare the polymerizable precursor solution and the hydrogel did not contain the PS 2DPC array.

**Figure 3:**
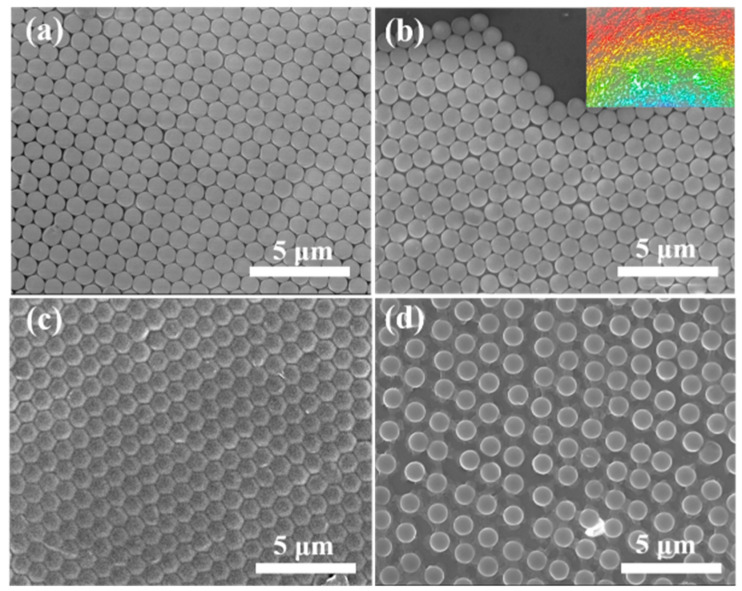
(**a**,**b**) SEM images of 2DPC array prepared from ~960 nm of PS microspheres on glass slide; Inset in (**b**) a photograph of the 2DPC array taken under illumination with a flashlight below at an angle of ~75° to the normal. The PS microspheres arrange orderly and closely on the glass slide and form a 2D monolayer array with a vivid rainbow color. (**c**) SEM image of the 2DPC hydrogel prepared initially. (**d**) SEM image of the 2DPC hydrogel equilibrated in PBS solution. The PS microspheres are still close-packed for the freshly prepared 2DPC hydrogel, while they become non-close-packed after equilibration in PBS solution due to the swelling of the hydrogel.

SEM was used to characterize the surface morphology of 2DPC and the photonic hydrogels. Figure 3a shows an SEM image of the 2DPC array prepared by ~960 nm of PS microspheres on the glass substrate, presenting a hexagonal close-packed structure and ordered monolayer array (Figure 3b). The photograph of the 2DPC array displays a bright iridescent color (Figure 3b, inset). The SEM image of the initially prepared 2DPC hydrogel (Figure 3c) shows that the 2DPC array still maintained the periodically close-packed structure well after photopolymerization and the vivid rainbow color (not shown). For the 2DPC hydrogel equilibrated in PBS solution, the PS microspheres were still arranged in a periodic structure, but in a non-close-packed hexagonal array (Figure 3d) due to the swelling of the hydrogel.

### 3.2. Response Mechanism of the Aptasensor

**Scheme 2:**
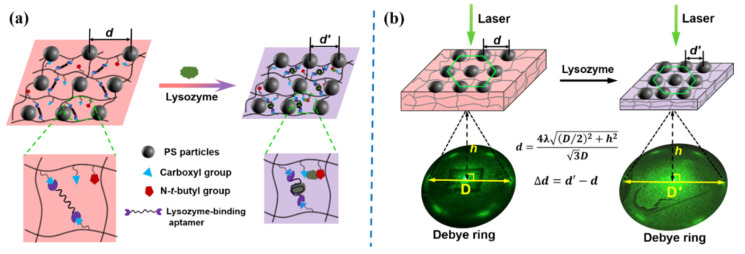
(**a**) The response mechanism of the photonic hydrogel aptasensor toward lysozyme. The specific binding between the aptamer and lysozyme induces its conformational change from a single-strand stretch to a G-quadruplex structure. This shortens the distance of the polymer chains and increases the cross-linking density of the hydrogel, leading to its shrinkage. In addition, the adjacent carboxyl groups and the hydrophobic N-tert butyl groups simultaneously interact with lysozyme based on the electrostatic interaction and hydrophobic groups interaction, which is also helpful to the shrinkage of the hydrogel. The hydrogel shrinkage induces the particle spacing (*d*) to decrease. (**b**) Measurement of Debye diffraction ring. The 2DPC embedded in the hydrogel network strongly diffracts light in the forward direction upon illumination by a laser along its array normal, generating a Debye ring on the screen below. The Debye ring diameter (*D*) can be measured directly and used to calculate the particle spacing of 2DPC according to a formula, $d=4\;\lambda_{laser}\sqrt{{\left(D/2\right)}^{2}+h^{2}}/\left(\sqrt{3}D\right)$, in which *λ_laser_* is the laser wavelength, and *h* is the distance between the 2DPC plane and the screen below. Lysozyme induces the photonic hydrogel to shrink and its particle spacing decreases. The particle spacing changes (Δ*d*) can be obtained by measuring the Debye ring diameters of the photonic hydrogel before and after response toward lysozyme and the following calculation of d.

Scheme 2a proposes a response mechanism of the constructed photonic hydrogel aptasensor toward lysozyme. When the hydrogel aptasensor is exposed to lysozyme solution, the aptamers linked on the polymer chains specifically bind lysozyme and their initial single-stranded stretching conformation changes into G-quadruplex structure [51], shortening the distance between the polymer chains. The conformational change induced by the specific binding between the aptamer and lysozyme enables an increase in the cross-linking density of the hydrogel, resulting in its shrinkage. Furthermore, the adjacent carboxyl groups and the hydrophobic N-tert-butyl groups simultaneously interact with lysozyme molecules, which also is helpful to the shrinkage of the hydrogel [52,53,54]. Herein, in PBS solution of pH 7.40, the carboxyl groups are negatively charged, and lysozyme is positively charged because its isoelectric point is ~10.8. Therefore, lysozyme can be bound to the carboxyl anions through electrostatic interaction, and, meanwhile, its hydrophobic residues interact with the hydrophobic N-tert-butyl groups, leading to the contraction of the hydrogel [53]. Upon the cooperative actuation based on the multi-factors including conformational change of the aptamer, electrostatic interaction and the interaction between hydrophobic groups, the hydrogel volume decreases and a significant shrinkage occurs. As a result, the particle spacing (*d*) of the 2DPC embedded in the hydrogel aptasensor reduces.

The particle spacing can be obtained by measuring the diameter (*D*) of the Debye diffraction ring of the 2DPC in hydrogel, as shown in Scheme 2b. The periodically hexagonally arranged 2DPC diffracts light and projects on the bottom screen when it is illuminated by a laser along the array normal. The Debye diffraction ring diameter reflects the particle spacing of the 2DPC hydrogel and the particle spacing can be calculated according to the formula shown in Scheme 2b. Then, the particle spacing change (Δ*d*) will be acquired by recording the Debye ring diameters of the hydrogel aptasensor before and after exposure to lysozyme solution and the following calculation of *d*. The magnitude of the particle spacing change is related to the lysozyme content and can be used as sensing signal to quantitatively determine the lysozyme concentration in the testing solutions.

**Figure 4:**
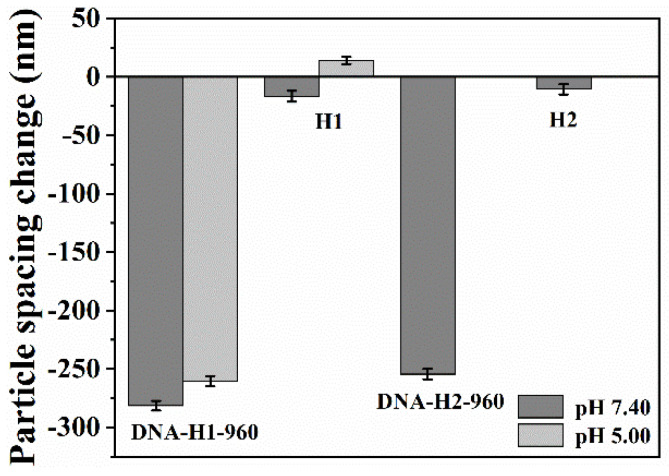
The particle spacing changes of various hydrogel films upon reaction with 500 μM lysozyme solutions. Compared with the hydrogels H1 and H2, the remarkable particle spacing decreases of the hydrogel sensors DNA−H1−960 and DNA−H2−960 with aptamers demonstrated the key actuation role of specific binding between the aptamer and lysozyme in the hydrogel shrinkage. Compared with DNA−H2−960 without N-tert-butyl groups, the larger particle spacing decrease of DNA−H1−960 showed the presence of the N-tert-butyl groups in the hydrogel network also facilitated the hydrogel shrinkage. The particle spacing change of DNA−H1−960 at pH 7.40 was larger than that at pH 5.00, showing the electrostatic interaction between carboxyl anions and lysozyme.

We prepared different photonic hydrogel aptasensors using ~960 nm of PS microspheres to prove the proposed response mechanism in Scheme 2a. The 2DPC hydrogels H1 and H2 were prepared by dripping 110 μL of precursor solutions with different compositions (Table 1), which were then modified by 100 μM lysozyme-binding aptamer to obtain different photonic hydrogel aptasensors, which were labelled as DNA−H1−960 and DNA−H2−960, respectively. Here, we used the photonic hydrogel aptasensor that was prepared by 100 μM aptamer because it showed the largest particle spacing decreases upon response to 500 μM lysozyme solution, as shown in Figure S2. All the above-prepared hydrogels were immersed in 500 μM lysozyme solution for 1 h, and the particle spacing changes are shown in Figure 4. The magnitudes of the particle spacing decrease for DNA−H1−960 and DNA−H2−960 were 281.4 ± 4.1 nm and 254.47 ± 4.4 nm, respectively. They were much larger than those of H1 and H2 (16.61 ± 4.7 nm and 10.56 ± 4.3 nm, respectively) without aptamers, proving the key role of the aptamer in the hydrogel shrinkage. This is because the modified aptamer in the hydrogel specifically bound lysozyme, and its conformational change drove the volume shrinkage of the hydrogel and the reduction of the microsphere spacing. In addition, the particle spacing decrease of DNA−H1−960 was larger than that of DNA−H2−960 (without TBAm) and much larger than that of H2 (without the aptamer and TBAm). It indicated that the simultaneous interactions between the negatively charged carboxyl groups, hydrophobic N-tert-butyl groups and lysozyme, also played roles in the hydrogel shrinkage. Obviously, the introduction of TBAm monomer helped the hydrogel to bind lysozyme. To further testify the electrostatic interaction between carboxyl anions and lysozyme, we monitored the particle spacing changes of DNA−H1−960 when exposed to 500 μM lysozyme PBS solution at pH 5.00, in which there were no negatively charged carboxyl groups. The particle spacing change of DNA−H1−960 at pH 5.00 PBS solution containing lysozyme was smaller than that at pH 7.40. This is because the carboxyl groups without a negative charge cannot bind lysozyme through electrostatic interaction. Furthermore, the swelled H1 at pH 5.00 lysozyme solution demonstrated the interaction between the N-tert-butyl groups and the hydrophobic residues on the surface of lysozyme. We also found that there was only a difference of ~27 nm between the particle spacing change of DNA−H1−960 and DNA−H2−960, showing that the shrinkage caused by the simultaneous interaction of carboxyl groups and N-tert-butyl with lysozyme was much smaller than that caused by the conformational change of the aptamer. Therefore, the specific binding of the aptamer dominantly actuated the hydrogel shrinkage. The above results demonstrated the multi-factors actuation mechanism of the constructed photonic hydrogel aptasensors for lysozyme. Furthermore, we also verified the response of the aptasensor to lysozyme by XPS characterization (Figure S3). The characteristic peaks at 227.02 eV and 163.33 eV were derived from the S2s and S2p, respectively, demonstrating the response of the photonic hydrogel aptasensor toward lysozyme. According to the largest particle spacing change, we selected DNA−H1−960 as the sensing film to study the detection performance of lysozyme.

### 3.3. Response Performance of DNA−H1−960 toward Lysozyme

**Figure 5:**
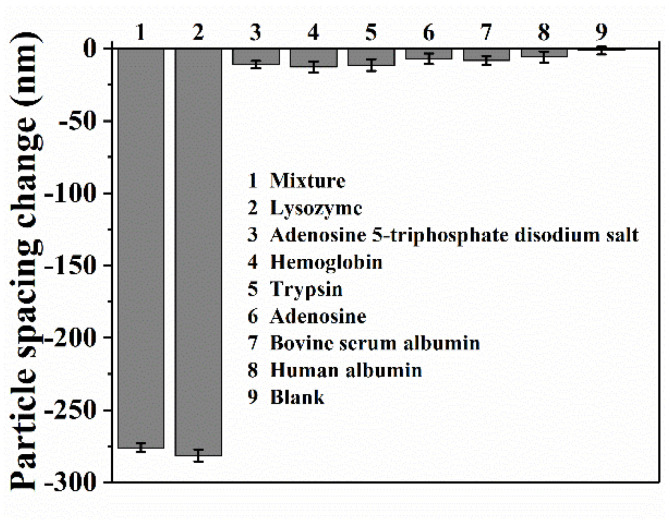
The particle spacing changes of the DNA−H1−960 after treatment with various solutions of 500 μM. The particle spacing decreases of the sensor in lysozyme and the mixture solution (1 Mixture, prepared by mixing the compounds 2–8 listed in this figure) were much higher than those in the other biomolecules’ solutions. The results showed the high detection selectivity and anti-interference of the DNA−H1−960 to lysozyme.

The response selectivity was first investigated by immersing the DNA−H1−960 photonic hydrogel aptasensors into 500 μM PBS solutions of lysozyme, adenosine 5−triphosphate disodium salt, hemoglobin, trypsin, adenosine, bovine serum albumin, human albumin and a mixture solution (Mixture) including the above-mentioned biomolecules for 1 h, respectively. In the mixture solution, the concentration of each compound was at 500 μM. The particle spacing changes are shown in Figure 5. The maximum particle spacing change of −281.4 ± 4.1 nm can be observed when the aptasensor was treated with lysozyme solution, while it was less than −12.67 ± 3.9 nm for other biomolecular solutions and the blank solution. It indicated that the constructed DNA−H1−960 had excellent selectivity for the detection of lysozyme, which can be attributed to the specific binding between the aptamer and lysozyme. Furthermore, the mixture solution also generated a significant particle spacing change of −276.02 ± 3.0 nm, demonstrating that the presence of other biomolecules did not affect the response of the DNA−H1−960 aptasensor toward lysozyme. The results proved that the constructed photonic hydrogel aptasensors had good anti-interference ability for detecting lysozyme.

**Figure 6:**
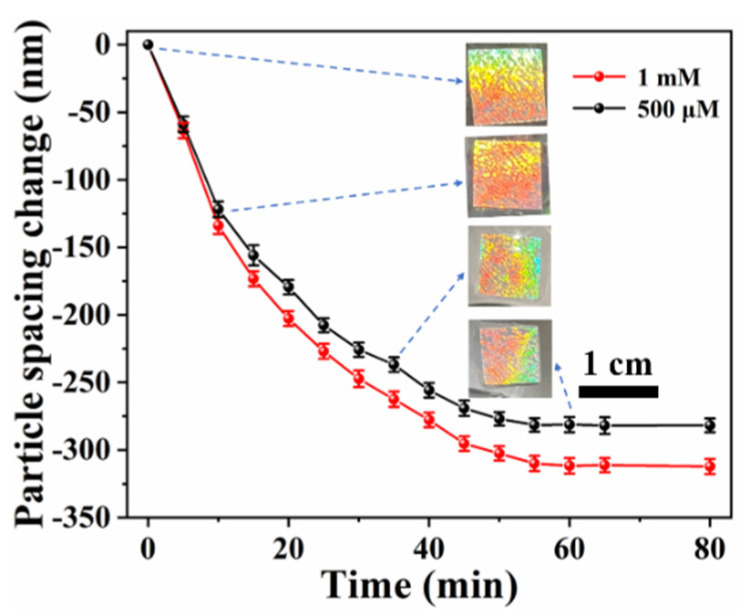
Time dependence of the particle spacing changes for DNA−H1−960 hydrogel aptasensor in 500 μM and 1 mM lysozyme solutions (Insets: the photographs of DNA−H1−960 at different response time). The scale bar “1 cm” represents the size of the photographs. The particle spacing decreased sharply within 10 min and achieved the response equilibrium at 60 min. The size of the hydrogel was obviously decreased along a longer reaction time.

To investigate the response time of DNA−H1−960 toward lysozyme, the aptasensor film was immersed in 500 μM and 1 mM lysozyme solution, and the particle spacing changes at different times were recorded. As shown in Figure 6, the particle spacing decreased sharply within 10 min. The magnitude of the particle spacing decrease gradually achieved the maximum of −281.25 ± 5.6 nm (500 μM) and −311.7 ± 5.7 nm (1 mM) at 60 min, and then tended to stability. Additionally, the photographs of the DNA−H1−960 hydrogel aptasensor at different times in 500 μM lysozyme solution (insets in Figure 6) also demonstrated the hydrogel’s shrinkage. Clearly, the size of the hydrogel decreased with increasing reaction time. Therefore, we can preliminarily judge whether the hydrogel responds to lysozyme by visual observation or measuring the size of the hydrogel.

**Figure 7:**
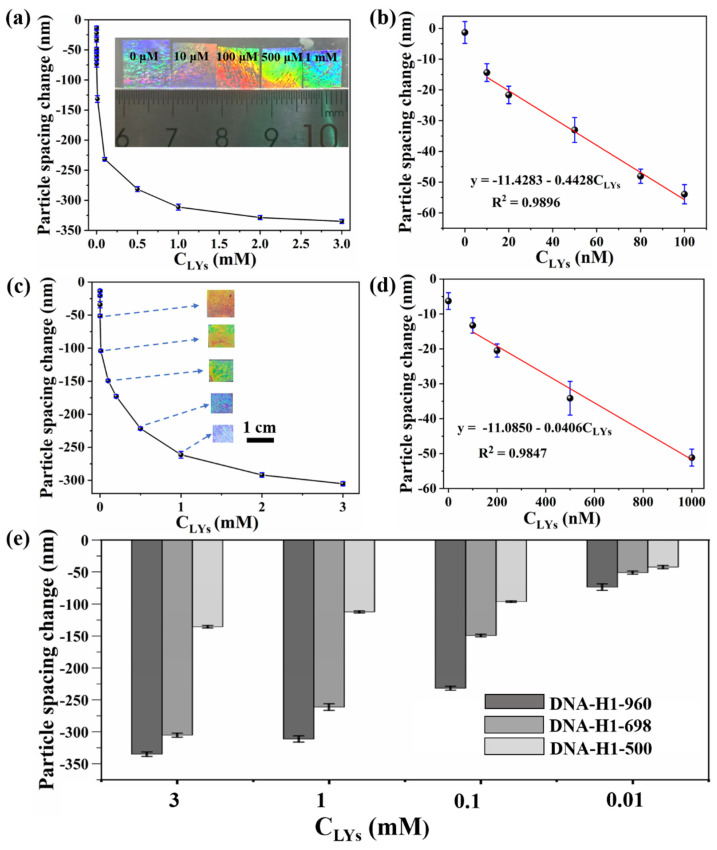
The particle spacing changes of the hydrogel aptasensors after reaction with different concentrations of lysozyme (C_LYs_): (**a**) DNA−H1−960; (**c**) DNA−H1−698; The insets are photographs of the corresponding aptasensors after achieving response equilibrium at different concentrations of lysozyme. The scale bar “1 cm” represents the size of the photographs. The linear relationship between the particle spacing changes and C_LYs_: (**b**) DNA−H1−960; (**d**) DNA−H1−698. (**e**) The particle spacing changes of DNA-H1 aptasensors fabricated from different diameters of PS microspheres upon exposure to different concentrations of lysozyme. The particle spacing changes increased with an increase in the lysozyme concentration and the limit of detection for DNA−H1−960 and DNA−H1−698 were 1.8 nM and 56.3 nM, respectively, indicating a more sensitive detection of lysozyme for DNA−H1−960. The comparison of the particle spacing changes at the same lysozyme concentrations demonstrated that the 2DPC hydrogel aptasensor prepared from larger diameter microspheres showed a clearer particle spacing change to lysozyme, which is more beneficial to the lysozyme detection.

Figure 7a shows the changes in particle spacing after the DNA−H1−960 aptasensor was exposed to different concentrations of lysozyme solution for 1 h. As the lysozyme concentration (C_LYs_) increased from 10 nM to 3 mM, the particle spacing change rapidly increased from −13.36 ± 2.9 nm to −335.1 ± 3.5 nm, and then gradually reached an equilibrium with the further increase of C_LYs_. The photographs of the hydrogel aptasensor after achieving response equilibrium at different lysozyme concentrations are shown in the insets of Figure 7a. Clearly, the hydrogel size gradually decreased with an increase in C_LYs_, indicating that the hydrogel shrank in the presence of lysozyme and underwent a larger shrinkage at higher concentrations. The particle spacing change of DNA−H1−960 showed a good linear relationship (R^2^ = 0.9896) when C_LYs_ was between 10−100 nM, as shown in Figure 7b. According to LoD = 3σ/k, the LoD of lysozyme was calculated to be 1.8 nM (Section 4 in the Supplementary Materials), in which σ is the standard deviation of blank measurements, and k is the slope of the linear relationship between C_LYs_ and the particle spacing change of aptasensors. However, the hydrogel H1 without the aptamers showed a significantly high LoD of 37.2 μM (Figure S4) compared with the DNA−H1−960, further demonstrating the decisive rule of the aptamer in this sensing platform. Additionally, the lysozyme detection based on photonic hydrogel DNA−H1−960 provided an excellent LoD compared with other detection method (Table S2) [55,56,57,58,59,60,61,62,63,64]. The results demonstrated that the constructed DNA−H1−960 can be a sensitive aptasensor for the detection of lysozyme.

Although the constructed DNA−H1−960 hydrogel aptasensor showed obvious volume shrinkage in lysozyme solutions at different concentrations, there was no obvious hydrogel color change, which can be observed by the naked eye due to the use of PS microspheres with a larger diameter. Thereupon, we chose PS microspheres of ~698 nm to prepare another aptasensor, in which other preparation conditions were same as DNA−H1−960. The newly prepared photonic hydrogel aptasensor was labeled as DNA−H1−698. 

The particle spacing changes of DNA−H1−698 at different concentration of lysozyme solutions after achieving response equilibrium are shown in Figure 7c. The magnitudes of the particle spacing decrease changed from −13.28 ± 2.2 nm to −305.21 ± 3.1 nm when C_LYs_ was in the range of 100 nM−3 mM. The insets of Figure 7c are optical photographs taken from an angle of ~75° along the normal of the DNA−H1−698 aptasensor after reaction with different concentrations of lysozyme solutions. The color of the DNA−H1−698 film was red upon exposure to 1 μM lysozyme solution, and gradually changed to green and violet as C_LYs_ increased. Simultaneously, the size of the hydrogel decreased, representing its shrinkage. Therefore, the photonic hydrogel aptasensor prepared from a smaller diameter of PS microspheres enabled the visual detection of lysozyme.

The magnitudes of the particle spacing decrease of DNA−H1−698 linearly increased against the C_LYs_ between 100−1000 nM (Figure 7d) and the LoD was found to be 56.3 nM (LoD = 3σ/k) (Section 4 in the Supporting Information). Compared with the results from DNA−H1−960, the linear range became wider, but the LoD was obviously higher. It is worth noting that the linear range of DNA−H1−698 was just conterminous to the linear range obtained from DNA−H1−960. It indicated that we can select a suitable photonic hydrogel aptasensor to determine the lysozyme concentration according to actual needs.

We found that in DNA−H1−960 there occurred a larger particle spacing decrease than in DNA−H1−698 at the same lysozyme concentration (Figure S5). This indicated that the particle spacing change of the photonic hydrogel aptasensors was related to the diameter of the microsphere used for preparation of 2DPC. To further explore the effect of the microsphere diameter on the particle spacing changes of the hydrogel aptasensors, we used the same method to prepare a DNA−H1−500 aptasensor with ~500 nm of PS microspheres. The LoD was also studied and found to be 97.0 nM (Figure S6), which is clearly higher than those from DNA−H1−960 and DNA−H1−698. In addition, we compared the particle spacing changes of these three photonic hydrogel aptasensors at the same lysozyme concentrations. The results shown in Figure 7e demonstrated that the particle spacing changes of the aptasensors prepared from microspheres with larger diameter were clearly larger than those of the aptasensors prepared from microspheres with smaller diameter, especially at higher lysozyme concentrations. To explain this phenomenon, we compared the particle spacings of these aptasensors after response toward 3 mM lysozyme solution with the used PS microspheres diameters. The particle spacings of DNA−H1−960, DNA−H1−698 and DNA−H1−500 after treatment with lysozyme were 1077.08 ± 2.4 nm, 820.31 ± 1.9 nm and 622.41 ± 1.4 nm, respectively, which were ~120 nm differences with the corresponding PS particle diameter. In consideration of the existence of hydrogel polymer chains and the negative charge repulsion between PS microspheres, we speculate that the particle spacings of these aptasensors after response toward lysozyme had decreased to a limit and were gradually close to the used PS microsphere diameter. Therefore, the 2DPC hydrogel aptasensor prepared from microspheres with a larger diameter showed a clearer particle spacing change in response to lysozyme, which is more beneficial to lysozyme detection. 

### 3.4. Detection of Lysozyme in Human Serum

We selected the DNA−H1−960 photonic hydrogel aptasensor to detect lysozyme in human serum. The detailed detection is shown in Section 6 in the Supplementary Materials. First, the aptasensors were immersed in the human serum testing solutions containing 20 nM, 50 nM and 80 nM of lysozyme for 1 h and the particle spacing changes were recorded. Then, the concentration of lysozyme in human serum was calculated according to the linear relationship y = −11.4283 − 0.4428C_LYs_ (Figure 7b) and compared with the spiked concentrations. As shown in Table 2, the recoveries for the detection of lysozyme in human serum were between 99.94–106.90% and the relative standard deviations (RSD) were 0.64–1.05%. The accurate detection of lysozyme in the commercially available human serum is based on the selected aptamer with the specific bases sequence. It is reported that the aptamer shows a low dissociation constant (*K_d_* = 31 nM) for lysozyme, implying a high affinity to lysozyme [65]. Therefore, the constructed photonic hydrogel aptasensor can be well applied to determine the concentration of lysozyme in human serum.

## 4. Conclusions

An aptamer-modified photonic hydrogel biosensor was constructed for the detection of lysozyme in human serum. The prepared aptasensor was actuated to generate a sensitive response toward lysozyme through multi-factors including the specific binding between the aptamer and lysozyme, the electrostatic interaction between carboxyl anion and lysozyme and the interaction between N-tert-butyl and hydrophobic residues of lysozyme. These multi-factors cooperatively increased the hydrogel cross-linking density and induced the aptasensor to shrink. The shrinkage of the hydrogel caused the particle spacing of the 2DPC embedded in the hydrogel network to decrease. The particle spacing change was acquired by measuring the Debye diffraction ring of the 2DPC, achieving the detection of lysozyme. The constructed hydrogel aptasensor can detect lysozyme with high selectivity, anti-interference and sensitivity with a LoD of 1.8 nM. In addition, we prepared different lysozyme-sensitive 2DPC hydrogel aptasensors by changing the PS microspheres’ diameter and achieved the visual detection of lysozyme. Furthermore, we studied the influence of the PS microspheres’ diameter on the particle spacing changes. The results demonstrated that the hydrogel aptasensors prepared from larger microspheres showed more obvious particle spacing changes and higher sensitivity. The prepared hydrogel aptasensor can reliably and accurately detect lysozyme in human serum with recoveries of 99.94–106.90% and RSD of 0.64–1.05%, which provides a simple, colorimetric and label-free method for the detection of lysozyme. It also establishes a universal approach for constructing photonic hydrogel aptasensors for other target analytes which can be bound to aptamers with specific sequences.

## Figures and Tables

**Table 1:** Compositions of monomer solutions of different photonic hydrogels *.

Sample #	Total (g)	AAm (g)	AAc (g)	Bis (g)	TBAm (g)	H_2_O (g)
H1	15	1.20	0.45	0.045	0.16	13.15
H2					--	13.31

* 20 µL of Irgacure 2959 in DMSO (33%, *w*/*v*) was added to 1 mL of monomer solution to prepare the polymerizable precursor solution. The aptamer solution of 100 μM was used to prepare the corresponding aptasensor. **#** represents the sample number.

**Table 2:** The detection of lysozyme in human serum.

Sample	Added (nM)	Found (nM)	Recovery %	RSD % (*n* = 3)
Human serum	20	21.37	106.90	0.64
	50	49.97	99.94	0.76
	80	82.96	103.70	1.05

## Data Availability

Not applicable.

## Supplementary Materials

The following supporting information can be downloaded at: https://www.mdpi.com/article/10.3390/bios12080662/s1, Figure S1: Measurement of Debye Diffraction Ring; Figure S2: The Effect of the Modified-Aptamer Concentration on the Particle Spacing Changes; Figure S3: XPS Characterization; Figure S4: Calculation of LoD; Figure S5: The Particle Spacing Changes of Aptasensors at Different Lysozyme Concentrations; Figure S6: Sensitivity of DNA-H1-500 for Detecting Lysozyme; Table S1: The Raw Data of DNA-H1-960 for the Detection of Lysozyme in Human Serum; Table S2: Detection Performance Comparison of Different Sensors for Lysozyme Detection.
