# Supplementary material for: Multi-Factors Cooperatively Actuated Photonic Hydrogel Aptasensors for Facile, Label-Free and Colorimetric Detection of Lysozyme
Source: Biosensors (Basel). 2022 Aug 20;12(8):662. doi: 10.3390/bios12080662 (PMC9406194; doi:10.3390/bios12080662)
Supplement: Supplementary file 1 [file biosensors-12-00662-s001.zip › biosensors-1841995-supplementary.pdf]

Supplementary Information

Multi-Factors Cooperatively Actuated Photonic Hydrogel Aptasensors for Facile, Label-Free and Colorimetric Detection of Lysozyme

Peiyan Shen[1,‡], Yuqing Shi[1,‡], Ran Li[1], Bo Han[1], Haojie Ma[1], Xueyan Hou[1], Yuqi Zhang[1,*] and Lei Jiang[2,*]

[1] *Key Laboratory of New Energy & New Functional Materials, Shaanxi Key Laboratory of Chemical Reaction Engineering, College of Chemistry and Chemical Engineering, Yan'an University, Yan'an, Shaanxi 716000, China*

[2] *Technical Institute of Physics and Chemistry, Chinese Academy of Sciences, Beijing 100190, China*

[*] *Corresponding author (email: yqzhang@yau.edu.cn; jianglei@mail.ipc.ac.cn)*

1. Measurement of Debye Diffraction Ring

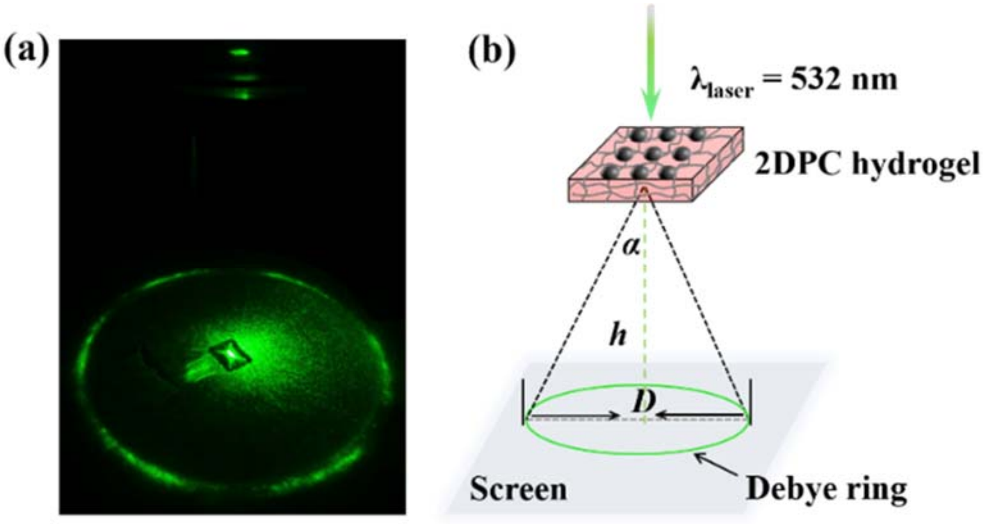

Figure S1. (**a**) A photograph and (**b**) measurement of Debye diffraction ring. The particle spacing (*d*) of the 2DPC hydrogel can be calculated according to a formula $d = 4\lambda\sqrt{(D/2)^2 + h^2}/(\sqrt{3}D)$, in which λ is the laser light wavelength, D is the Debye ring diameter, and h is the distance between the 2DPC array and the bottom screen.

2. The Effect of the Modified-Aptamer Concentration on the Particle Spacing Changes

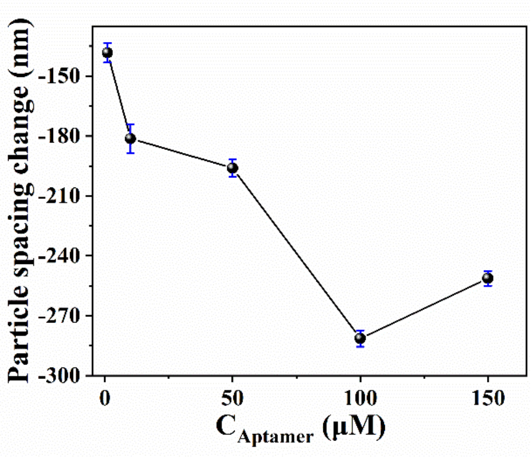

Figure S2. The particle spacing changes for the different concentrations of aptamer functionalized 2DPC hydrogel sensors under the same preparation conditions as sample DNA-H1-960 in the 500 μM lysozyme solution.

3. XPS Characterization

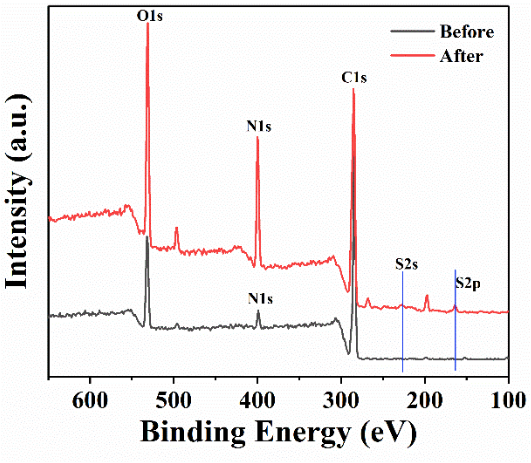

Figure S3. The XPS of DNA-H1-960 before and after response to lysozyme. The peaks at 227.02 eV and 163.33 eV were derived from the S2s and S2p, respectively, demonstrating the response of the photonic hydrogel aptasensor toward lysozyme.

4. Calculation of LoD

The LoD of the 2DPC hydrogel aptasensors was calculated from the formula LoD = $3\sigma/k$, in which σ is the standard deviation of blank measurements, and k is the slope of the linear relationship between C_{LYs} and the particle spacing change of the aptasensors.

$$\sigma = \sqrt{\frac{\sum_{i=1}^{n}(x_i - \bar{x})^2}{n-1}} \quad (1)$$

(1) DNA-H1-960

Sample	1	2	3	4	5	Average
Particle spacing change (nm)	−1.235	−1.641	−1.134	−1.615	−1.028	−1.325

For the particle spacing change measurements, the five blank and their average are shown in the above Table.

Then σ was calculated as follows:

$$\sigma = \sqrt{\frac{0.0081 + 0.0999 + 0.0365 + 0.0841 + 0.0882}{5-1}} = 0.2814$$

The linear correlation of the particle spacing change and lysozyme concentration is y = -11.4283 – 0.4428C_{LYs} (Figure 7b). Thus the LoD can be calcultated as follows:

$$LoD_{\text{DNA-H1-960}} = \frac{3\sigma}{k} = \frac{3 \times 0.2814}{0.4428} = 1.8 \text{ nM}$$

(2) DNA-H1-698

Sample	1	2	3	4	5	Average
Particle spacing change (nm)	-6.823	-5.809	-6.759	-5.208	-6.934	-6.307

Then σ was calculated as follows:

$$\sigma = \sqrt{\frac{0.2663 + 0.2480 + 0.2043 + 1.2078 + 0.3931}{5-1}} = 0.7615$$

The linear correlation of the particle spacing change and lysozyme concentration is y = -11.4283 – 0.0406C_{LYs} (Figure 7d). Thus the LoD was as follows:

$$LoD_{\text{DNA-H1-698}} = \frac{3\sigma}{k} = \frac{3 \times 0.7615}{0.0406} = 56.3 \text{ nM}$$

(3) Hydrogel H1

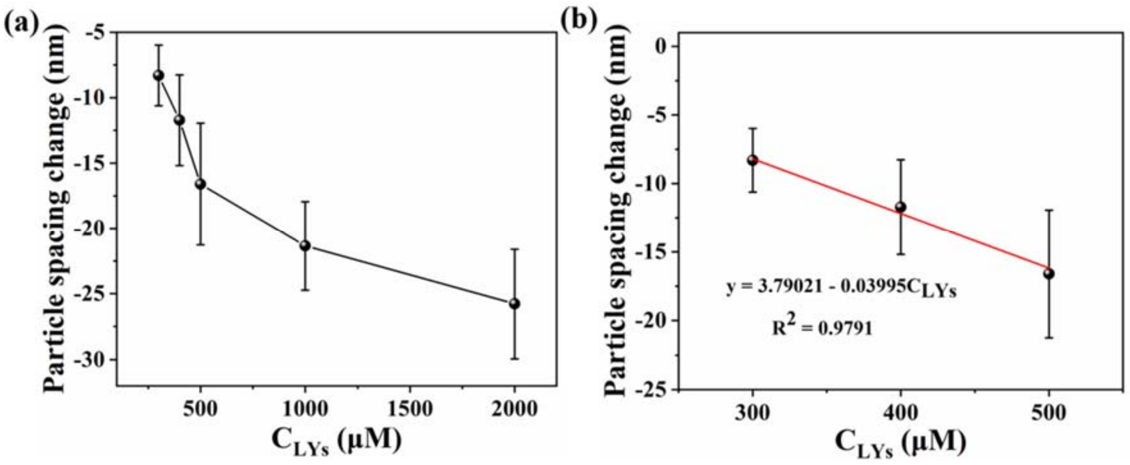

Figure S4. (**a**) The particle spacing changes of the H1 after reaction with different concentrations of lysozyme (C_{LYs}): (**b**) The linear relationship between the particle spacing changes and C_{LYs}.

5. The Particle Spacing Changes of Aptasensors at Different Lysozyme Concentrations

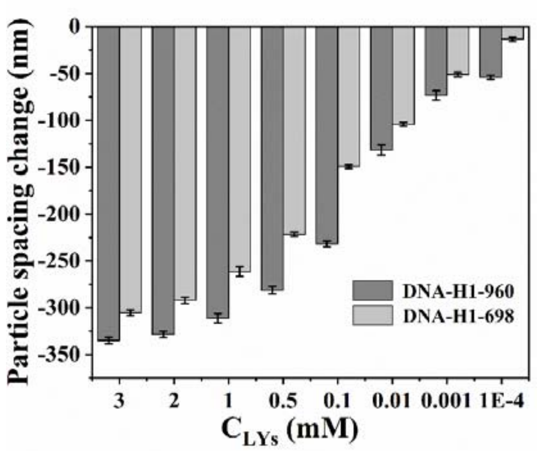

Figure S5. The particle spacing changes of DNA-H1 hydrogel aptasensors fabricated from different diameters of PS microspheres upon exposure to different concentrations of lysozyme solution. Clearly, the particle spacing changes of DNA-H1-960 are larger than those of DNA-H1-698 at same lysozyme concentration. It indicated that the photonic hydrogel aptasensor fabricated from larger PS microspheres occurred clearer shrinkage, which is helpful to the detection of lysozyme.

6. Sensitivity of DNA-H1-500 for Detecting Lysozyme

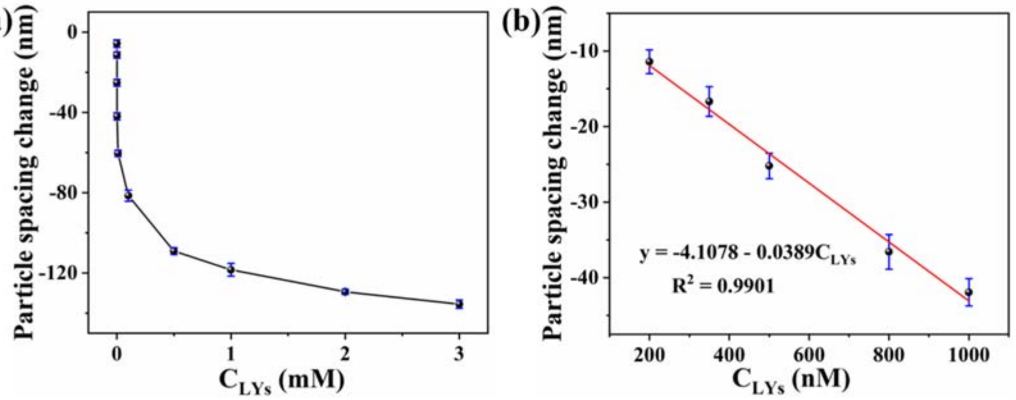

Figure S6. (**a**) The particle spacing changes of DNA-H1-500 when exposed to different concentrations of lysozyme solutions (C_{LYs}) and (**b**) the linear relationship over a range of 200-1000 nM. The particle spacing decreased with an increase in the lysozyme concentrations and there is a good linear correlation between the particle spacing changes and the lysozyme concentrations. The limit of detection (LoD) was found to be 97.0 nM (LoD = 3σ/k, in which σ is the standard deviation of blank measurements, and k is the slope of the linear relationship between C_{LYs} and the particle spacing change of aptasensors).

7. Detection of Lysozyme in Human Serum

The response of DNA-H1-960 was validated in human serum. The commercial human serum was diluted 10-fold with PBS solution. Then, the known concentrations lysozyme was spiked into the diluted human serum as testing solutions. The detection of lysozyme in the testing solution was similar to that in PBS solution. The particle spacing of DNA-H1-960 was recorded before and after in response to a testing solution to evaluate the practical applicability of DNA-H1-960 (Table S1).

The raw data for detecting lysozyme in human serum are provided in Table S1.

Table S1. The Raw Data of DNA-H1-960 for the Detection of Lysozyme in Human Serum (n=3).

Samples	Testing solution containing lysozyme concentration (nM)	Initial particle spacing (d)	Particle spacing after reaction with testing solution (d')	Particle spacing change(Δd=d-d')	Found **C_{LYs}** * (nM)
Human serum	20	d=1403.522<br>s=4.046398	d'=1382.647<br>s=2.281306	Δd=-20.875<br>s=3.284638	21.33
		d=1407.023<br>s=3.935175	d'=1386.199<br>s=2.30601	Δd=-20.824<br>s=3.225158	21.22
		d=1397.428<br>s=2.616653	d'=1376.457<br>s=3.523263	Δd=-20.971<br>s=3.103245	21.55
	50	d=1392.718<br>s=2.730183	d'=1359.301<br>s=4.86	Δd=-33.417<br>s=3.941668	49.66
		d=1409.098<br>s=2.408436	d'=1375.308<br>s=1.762199	Δd=-33.79<br>s=2.110203	50.50
		d=1406.269<br>s=3.796502	d'=1372.817<br>s=2.141248	Δd=-33.452<br>s=3.082075	49.74
	80	d=1414.956<br>s=4.729316	d'=1366.736<br>s=3.548515	Δd=48.214<br>s=4.180813	83.08
		d=1405.67	d'=1357.063	Δd=-48.607	83.96

	s=2.939129	s=2.734192	s=2.838511	
	d=1437.563	d'=1389.894	Δd=47.669	81.84
	s=4.014542	s=4.326105	s=4.173232	

*The found value of C_{LYs} was calculated from the linear relationship y = -11.4283 – 0.4428C_{LYs}.

8. Table S2. Detection Performance Comparison of Different Sensors for Lysozyme Detection

Detection Method	Materials	Linear range	LoD	Ref.
Debye ring measurement	2DPC hydrogel	10 – 100 nM	1.8 nM	This work
Fluorometry	Au nanoparticles (AuNPs)	1 – 25 nM	10 nM	[1]
Fluorometry	Silver nanocluster	2 – 25nM	5.6 nM	[2]
Fluorimetry	Grapheme oxide	50 – 300 nM	11 nM	[3]
Colorimetry	Ag nanoparticles	3 – 150nM	1.5 nM	[4]
Colorimetry	CdTe/AuNPs	4 – 60nM	4.0nM	[5]
Colorimetry	DIC-Au NPs	100 – 800nM	8.7 nM	[6]
Colorimetry	AuNPs	10 –200 nM	8.7 nM	[7]
Colorimetry	Cu/Au NPs	100 nM – 1 mM	60 nM	[8]
Colorimetry	AuNPs	5 – 50 nM	0.1 nM	[9]
Colorimetry	AuNPs	4.4 – 200 nM	4.4nM	[10]

9. References

[1] Gu, P.; Liu, X.; Tian, Y.; Zhang, L.; Huang, Y.; Su, S.; Feng, X.; Fan, Q.; Huang, W. A Novel Visible Detection Strategy for Lysozyme Based on Gold Nanoparticles and Conjugated Polymer Brush. S*ens. Actuators B Chem.* **2017**, *246*, 78–84.

[2] Ardekani, L.S.; Moghadam, T.T.; Thulstrup, P.W.; Ranjbar, B. Design and Fabrication of a Silver Nanocluster-Based Aptasensor for Lysozyme Detection. *Plasmonics* **2019**, *14*, 1765–1774.

[3] Xie, Y.; An, J.; Shi, P.; Ye, N. Determination of Lysozyme by Graphene Oxide–Polyethylene Glycol-Based Fluorescence Resonance Energy Transfer. *Anal. Lett.* **2017**, *50*, 148–160.

[4] Shrivas, K.; Nirmalkar, N.; Deb, M.K.; Dewangan, K.; Nirmalkar, J.; Kumar, S. Application of Functionalized Silver Nanoparticles as a Biochemical Sensor for Selective Detection of Lysozyme Protein in Milk Sample. *Spectrochim. Acta A* **2019**, *213*, 127–133.

[5] Chen, L.; Xia, N.; Li, T.; Bai, Y.; Chen, X. Aptasensor for Visual and Fluorometric Determination of Lysozyme Based on the Inner Filter Effect of Gold Nanoparticles on CdTe Quantum Dots. *Microchi Acta* **2016**, *183*, 2917–2923.

[6] Kasibabu, B.S.B.; Bhamore, J.R.; D'souza, S.L.; Kailasa, S.K. Dicoumarol Assisted Synthesis of Water Dispersible Gold Nanoparticles for Colorimetric Sensing of Cysteine and Lysozyme in Biofluids. *RSC Adv.* **2015**, *5*, 39182–39191.

[7] Li, J.; Mu, X.; Chan, K.-C.; Ko, C.-C.; Li, M.-J. Sensitive Determination of Lysozyme by Using a Luminescent and Colorimetric Probe Based on the Aggregation of Gold Nanoparticles Induced by an Anionic Ruthenate(II) Complex. *Microchim. Acta* **2018**, *185*, 428.

[8] Lou, T.; Qiang, H.; Chen, Z. Core-Shell Cu@Au Nanoparticles-Based Colorimetric Aptasensor for the Determination of Lysozyme. *Talanta* **2017**, *163*, 132–139.

[9] Wang, S.; Hu, X.; Tan, L.; Liao, Q.; Chen, Z. Colorimetric Detection of Lysozyme Based on Its Effect on the Growth of Gold Nanoparticles Induced by the Reaction of Chloroauric Acid and Hydroxylamine. *Microchim. Acta* **2016**, *183*, 3135–3141.

[10] Yao, X.; Ma, X.; Ding, C.; Jia, L. Colorimetric Determination of Lysozyme Based on the Aggregation of Gold Nanoparticles Controlled by a Cationic Polymer and an Aptamer. *Microchim. Acta* **2016**, *183*, 2353–2359.
